# Candidate Gene Study of *TRAIL* and *TRAIL Receptors*: Association with Response to Interferon Beta Therapy in Multiple Sclerosis Patients

**DOI:** 10.1371/journal.pone.0062540

**Published:** 2013-04-29

**Authors:** Carlos López-Gómez, Almudena Pino-Ángeles, Teresa Órpez-Zafra, María Jesús Pinto-Medel, Begoña Oliver-Martos, Jesús Ortega-Pinazo, Carlos Arnáiz, Cristina Guijarro-Castro, Jezabel Varadé, Roberto Álvarez-Lafuente, Elena Urcelay, Francisca Sánchez-Jiménez, Óscar Fernández, Laura Leyva

**Affiliations:** 1 Research Laboratory. Clinical Neurosciences Institute, Hospital Regional Universitario Carlos Haya, Málaga, Spain; 2 Department of Molecular Biology and Biochemistry, University of Málaga, Málaga, Spain; 3 Department of Neurology. Clinical Neurosciences Institute, Hospital Regional Universitario Carlos Haya, Málaga, Spain; 4 Department of Neurology, Hospital Universitario 12 de Octubre, Madrid, Spain; 5 Multiple Sclerosis Unit, Hospital Clínico San Carlos, IdISSC, Madrid, Spain; 6 Centro de Investigación Biomédica en Red de Enfermedades Raras (CIBERER), Málaga, Spain; 7 Red Española de Esclerosis Múltiple (REEM RD 07/0060), Málaga, Spain; Institute Biomedical Research August Pi Sunyer (IDIBAPS) - Hospital Clinic of Barcelona, Spain

## Abstract

*TRAIL* and *TRAIL Receptor* genes have been implicated in Multiple Sclerosis pathology as well as in the response to IFN beta therapy. The objective of our study was to evaluate the association of these genes in relation to the age at disease onset (AAO) and to the clinical response upon IFN beta treatment in Spanish MS patients. We carried out a candidate gene study of *TRAIL, TRAILR-1, TRAILR-2, TRAILR-3* and *TRAILR-4* genes. A total of 54 SNPs were analysed in 509 MS patients under IFN beta treatment, and an additional cohort of 226 MS patients was used to validate the results. Associations of rs1047275 in *TRAILR-2* and rs7011559 in *TRAILR-4* genes with AAO under an additive model did not withstand Bonferroni correction. In contrast, patients with the *TRAILR-1 rs20576-CC* genotype showed a better clinical response to IFN beta therapy compared with patients carrying the *A*-allele (recessive model: p = 8.88×10^−4^, p_c_ = 0.048, OR = 0.30). This SNP resulted in a non synonymous substitution of Glutamic acid to Alanine in position 228 (*E228A*), a change previously associated with susceptibility to different cancer types and risk of metastases, suggesting a lack of functionality of TRAILR-1. In order to unravel how this amino acid change in TRAILR-1 would affect to death signal, we performed a molecular modelling with both alleles. Neither TRAIL binding sites in the receptor nor the expression levels of TRAILR-1 in peripheral blood mononuclear cell subsets (monocytes, CD4+ and CD8+ T cells) were modified, suggesting that this SNP may be altering the death signal by some other mechanism. These findings show a role for *TRAILR-1* gene variations in the clinical outcome of IFN beta therapy that might have relevance as a biomarker to predict the response to IFN beta in MS.

## Introduction

Multiple Sclerosis (MS) is a chronic, inflammatory, demyelinating and neurodegenerative disease of the Central Nervous System (CNS) in which immunomodulatory therapies are only partially effective.

The typical course of MS is characterized by symptom exacerbations followed by periods of remission. Nevertheless, the majority of patients will present disability progression, independently of exacerbations in the long run. It has been shown that early treatment with disease modifying drugs is the best strategy to reduce disease activity and slow progression of disability in MS [1], [2] **.** Recombinant interferon (IFN) beta is one of the most widely used first line therapy in MS. It reduces the number of relapses and brain magnetic resonance imaging activity and delays disability progression in relapsing/remitting (RR) MS [3], [4]. Nevertheless, up to 50% of patients treated with IFN beta continue experiencing relapses and/or worsening disability [5]. Hence, there are a large number of patients receiving, which is probably, a suboptimal treatment, making reliable biomarkers associated with the response to IFN beta therapy especially welcomed in the clinical practice. Several studies have searched for allelic variants associated with the response to IFN beta treatment in MS [6], [7], [8], [9], [10], [11], [12], [13], [14], [15], but, to the best of our knowledge, none of them has deeply analysed the genes of *TRAIL* and its four membrane receptors with regards to this response.

TNF Related Apoptosis Inducing Ligand (TRAIL) [16] is a type II transmembrane protein, belonging to the TNF/nerve growth factor superfamily, capable of inducing apoptosis in susceptible cells through interaction with its receptors TRAILR-1 and TRAILR-2. Two other cell-bound receptors - TRAILR-3 and TRAILR-4 - and a soluble receptor - Osteoprotegerin - do not contain functional death domains and act as decoy receptors for TRAIL. It is thought that the balance between death and decoy receptors underlies the sensitivity to TRAIL-induced apoptosis in different cell types [17].

TRAIL plays immunosuppressive, immunoregulatory and immune-effector functions, and it is involved in the pathogenesis of MS as well as in other autoimmune diseases [18], [19], although its precise role is not completely understood. Besides, TRAIL has been shown to be induced by IFN beta in T cells [20], [21], Natural Killer cells [22] and monocytes [23], and its mRNA level has been proposed as a response marker for IFN beta treatment [24]. We have previously reported the effects of SNPs in *TRAIL* and *TRAIL-Receptors* genes on MS susceptibility [25]. In the present work, we examined the potential role of polymorphisms in the genes encoding *TRAIL* and its four surface receptors on age at onset (AAO) and response to IFN beta therapy in MS. In addition, functional implications of the exonic SNP rs20576 in *TRAILR-1* were tested through an *in silico* molecular modelling, and through comparison of TRAILR-1 expression levels in peripheral blood mononuclear cell subsets in MS patients stratified by the rs20576 genotype.

## Results

### Genotyping

As previously described [25], from the 59 SNPs selected, three were discarded from the study because of technical problems in the manufacturing process (rs3136597, rs13257094 and rs4242387) and two other SNPs were discarded because of deviations from Hardy-Weinberg Equilibrium (rs3181143 and rs12545733). Genotype distribution of the remaining 54 SNPs in the original and in the validation cohorts is shown in Table S1, and genotype distribution stratified by IFN beta response is shown in Table S2.

### Age at Disease Onset

Two SNPs (rs1047275 in *TRAILR-2* and rs7011559 in *TRAILR-4*) with p values lower than 0.05 were found in the discovery cohort, but none of them were successfully replicated in the validation cohort (Table 1).

**Table 1:** Polymorphisms significantly associated with age at disease onset in the original cohort.

SNP ID/Gene	Original Cohort		Validation Cohort		Joint Analysis	
	Kruskal-Wallis	Mann-Whitney	Kruskal-Wallis	Mann-Whitney	Kruskal-Wallis	Mann-Whitney
rs1047275 *TRAILR-2*	p = 0.032	p = 0.009	p = 0.432	p = 0.363	P>0.1	p>0.1
rs7011559 *TRAILR-4*	p = 0.040	p = 0.012	p = 0.240	p = 0.252	p = 0.023	p = 0.009

Abbreviations: SNP ID, SNP identification; Mann-Whitney test corresponds to the comparisons between GG vs C/− genotypes for rs1047275 and GG vs A/− genotypes for rs7011559.

### Response to IFN Beta Therapy

The SNP rs20576 in exon 5 in *TRAILR-1,* which showed a trend in the original cohort (recessive model: p = 0.056, OR = 0.41), was significantly associated in the validation cohort (recessive model: p = 4.83×10^−3^, OR = 0.19) and it reached the Bonferroni level of significance in the joint analysis (recessive model: p = 8.88×10^−4^, pc = 0.048, OR = 0.30) (Table 2). The frequencies of the heterozygous genotype did not change between responders and nonresponders, indicating that the concurrence of the two mutant alleles present in the homozygous mutant genotype would be mandatory to produce an effect on IFN beta response. The borderline significance in the allelic comparison of responders and non-responders [p = 0.07; OR (95% CI) = 0.8 (0.62–1.03)] is due to the low minor allele frequency (MAF) of this polymorphism.

**Table 2:** Genotypes frequencies for rs20576 according to response to IFN beta in all patients and NABs-free patients.

	Response to IFN beta	A/− (%)	CC (%)	p value	OR (95% CI)
Original Cohort	Responders	201 (94.4)	12 (5.6)	0.06	0.41 (0.16–1.05)
	Non Responders	289 (97.6)	7 (2.4)		
Validation Cohort	Responders	90 (88.2)	12 (11.8)	0.005	0.19 (0.05–0.70)
	Non Responders	118 (97.5)	3 (2.5)		
Joint Analysis	Responders	291 (92.4)	24 (7.6)	8.88×10^−4^	0.30 (0.14–0.63)
	Non Responders	407 (97.4)	10 (2.4)		

P values and Odds Ratios under a recessive model. Abbreviations: A/−: wild type and heterozygous genotypes; OR (95% CI) = odds ratio with confidence interval at 95%.

To ensure that Neutralizing Antibodies (NABs) were not acting as confounding factors, we performed the same analysis restricted only to those patients with a negative test to NABs at both 12 and 24 months. We obtained NABs data at both times for a total of 488 patients, from which 350 were NAB-negative at both times. We excluded from the analysis those patients for which NAB status was only determined at either 12 or 24 months. The association of the *rs20576-CC* genotype with a good response to IFN beta showed a trend for significance in the joint analysis for the “NABs-free patients” after applying the highly conservative Bonferroni correction (p = 1.17×10^−3^, p_c_ = 0.063, OR = 0.13), as a consequence in the reduction of the sample size, which decreased the power to detect a genetic effect of OR = 1.5 at a 5% significance level in the joint cohort from 62.55% in the crude analysis to only 35.16% in the NABs-free population.

Additionally, we tested whether the disease course was affecting the analysis. Stratification of the patients only showed significant results in the RRMS patients as can be observed in Table S3 but, again, the genotype frequencies, fairly similar in both groups, as well as the OR, pointed to a loss of statistical power in the reduced group of SPMS patients with a sample size 4 times lower (n = 145 *versus* n = 587). In fact, stratifying the analysis by the disease course did not improve the results in the joint population (p = 3.15×10^−3^, OR = 0.29) indicating that this variable does not help to explain the different distribution of the CC genotype in the rs20576 among responders and non responders.

Finally, to test whether this genotype had a specific effect on the response to IFN beta treatment, the same analysis was performed in an independent cohort of Glatiramer Acetate-treated patients. In data obtained from 29 responders and 48 non responders to Glatiramer Acetate, we could not detect statistical differences in the distribution of rs20576 genotypes according to response to this drug for any of the genetic models assessed, as shown in Table S4.

### Molecular Modelling

**Figure 1:**
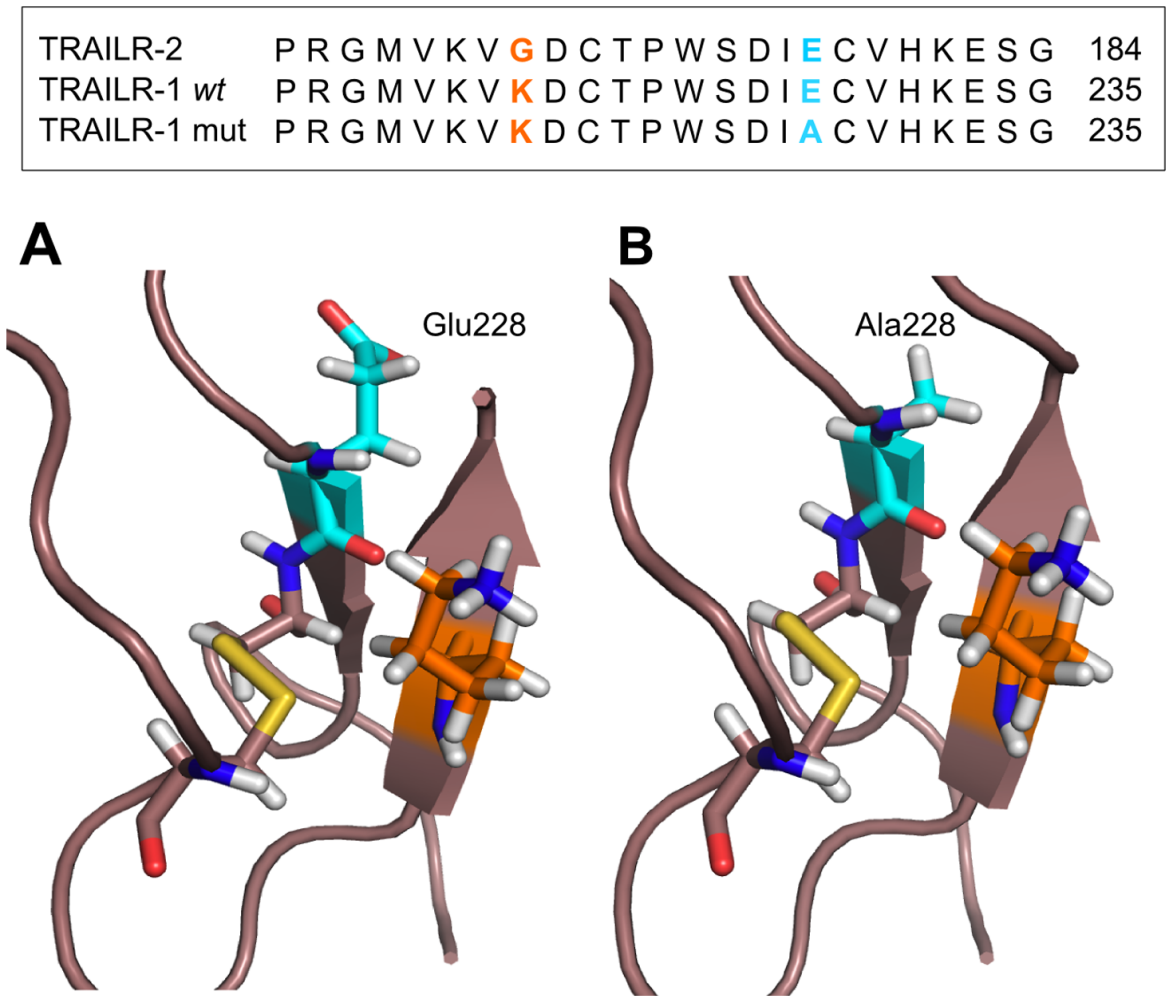
Alignment and secondary structure homology between TRAILR-2 and TRAILR-1. The high conservation in the amino acid sequence and secondary structure of both receptors has allowed us to use a slightly modified structure of TRAILR-2 in the molecular modelling.

**Figure 2:**
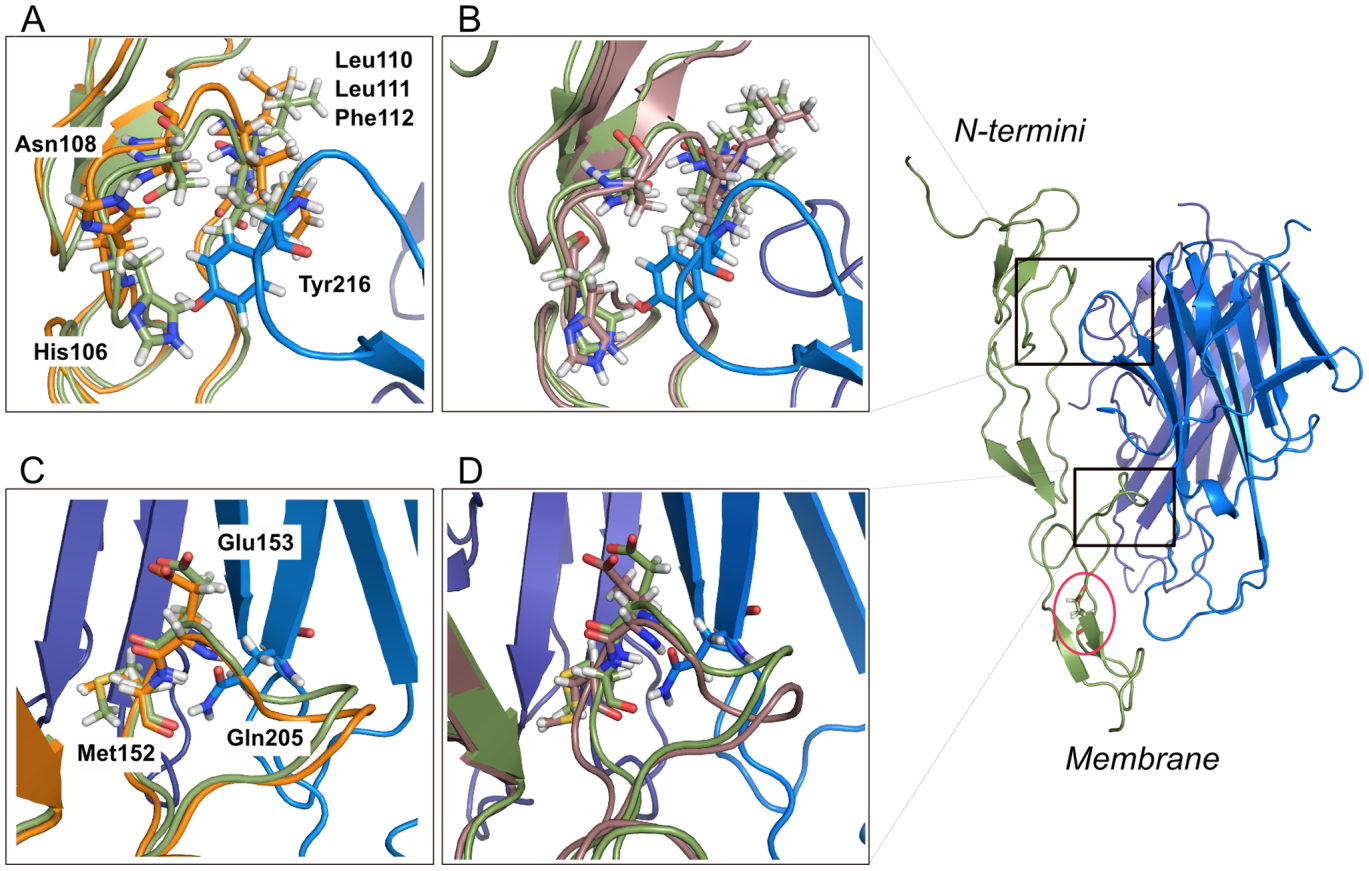
Molecular dynamics simulations of Glu228Ala SNP in TRAILR-1. A and C corresponds to the superimposed structures of the 50s and 90s loops respectively in the initial (green) and final (orange) model of the **wild type** of TRAILR-1 (Glutamatic acid at position 228). B and D corresponds to the superimposed structures of the loops in the initial (green) and final (brown) model of the **mutant type** of TRAILR-1 (Alanine at position 228).

The identity of TRAILR-1 and TRAILR-2 sequences and the high conservation of the secondary structure of both receptors allowed us to use a slightly modified version of the crystal structure of TRAILR-2 for this study. The polymorphism *E228A* in *TRAILR-1* has its counterpart in *E177* in *TRAILR-2*, and it is located in an extremely conserved region (as can be observed in Figure 1). When mapped into the structure of the receptor, E177 is not physically interacting with TRAIL. We have performed two molecular dynamic simulations, wild type and E177A structures, in order to study the putative effects of the polymorphism in the conformation of the death receptor. The RMSD values of the structures were calculated for the trajectories using the coordinates after the heating process as a reference. RMSD values for the wild-type complex show the stabilization of the structure around 2.5–2.8 Å after the first 2 ns of the simulation, whereas the variant complex quickly stabilizes to 2.2–2.3 Å. In order to evaluate the differences in the conformation of both complexes, two average structures comprising the last 500 ps of the simulations were generated and superimposed to the starting/initial structure in each case. Although the polymorphism is not located in any of the interaction surfaces between the receptor and TRAIL, our initial hypothesis meant to test the possibility of major conformational changes occurring in the structure due to the amino acid change. We paid special attention to the conserved 50′s and 90′s loops, which hold most of the key residues in the interaction with TRAIL. The 90′s loop hold the key residues M152 and E153, interacting with N205 in TRAIL, and their conformation remained very stable throughout the simulations in both our systems, as can be seen in Figure 2. Regarding the 50′s loop, several residues in it were arranged so their side chains created a hydrophobic pocket in which Y216 in TRAIL was accommodated. The residues in the 50′s loop kept a close conformation to the initial one, and Y216 side chain was oriented towards the hydrophobic pocket along the trajectory, as shown in Figure 2.

Hence, out of these computational results, there is no clear evidence that the polymorphism *E228A* prevents the formation of the complex between TRAILR-1 and TRAIL.

### Relative Expression of TRAILR-1

**Figure 3:**
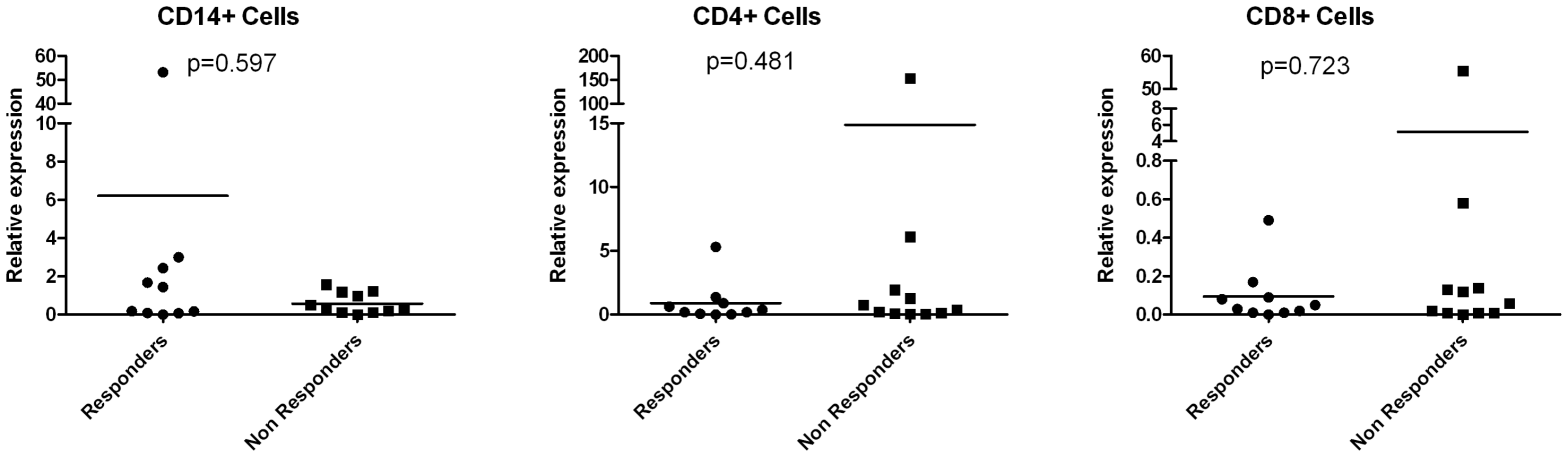
TRAILR-1 expression levels among Responders and Non Responders to IFN beta therapy, in different PBMC subsets. Expression level is represented as relative expression compared to the reference gene GAPDH, using the ΔΔCt method. TRAILR-1 expression levels between responder and non responder patients to IFN beta therapy in each of the assessed cell subsets were compared by a Mann Whitney test.

**Figure 4:**
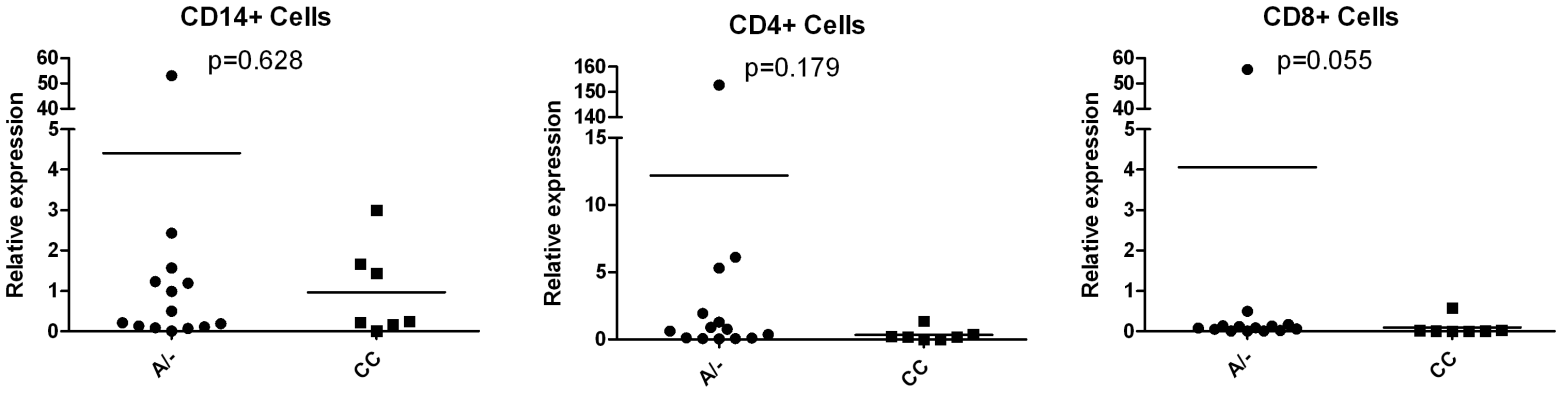
TRAILR-1 expression levels in MS patients stratified by the rs20576 genotype, in different PBMC subsets. Expression level is represented as relative expression compared to the reference gene GAPDH, using the ΔΔCt method. TRAILR-1 expression levels between patients with A/− and CC genotypes were compared by a Mann Whitney test.

In light of the negative results of the molecular modelling, we compared the expression levels of TRAILR-1 in the following PBMC subsets: monocytes (CD14+), CD4+ T cells and CD8+ T cells in MS patients. No significant differences were found in the relative expression between responders and non responders to IFN-beta, as shown in Figure 3. Stratification of the samples by the rs20576 genotype showed no significant differences between carriers of the wild type allele (AA and AC genotypes) and those with the CC genotype in any of these cell subsets, as can be observed in Figure 4.

## Discussion

Up to 50% of MS patients fail to response to IFN beta therapy, therefore biomarkers to predict the therapeutic response would permit to use this drug more appropriately, avoiding unnecessary exposure to it and making possible the rational use of the available monetary resources.

A previous study associated an early and sustained induction of TRAIL mRNA in unseparated PBMCs to good response to IFN beta therapy in MS [24] but, recently, some other authors have shown exclusive TRAIL mRNA induction in monocytes of clinical responders to IFN beta therapy [26]. They also showed an increase in TRAILR-3 expression in T cells from RRMS patients without IFN beta treatment, while those patients under this therapy presented a reduced level of expression of this decoy receptor. Supporting these data, a new study has reported TRAIL expression on granulocytes in all patients with RRMS after IFN beta stimulation [23]. On the contrary, TRAIL expression on monocytes was not achieved in all RRMS patients in response to this immunomodulatory drug [23]. Moreover, a recent article have reported that after T cell activation, TRAILR-2 is relocalized from cytoplasm to the cell surface in PHA-activated T cells, making them susceptible to TRAIL-induced apoptosis [27].

These data suggest, on the one hand, an enhancement of the proapoptotic effect of TRAIL on auto-reactive T cells as one of the mechanisms of action of IFN beta in MS, but, on the other hand, the induction of TRAIL on monocytes and granulocytes in response to IFN beta may contribute to the induction of apoptosis in these cells. Both types of cells are responsible of the breakdown of the blood–brain barrier (BBB). Besides, monocytes become activated and differentiate into macrophages when crossing the BBB acquiring potentially demyelinating capacities [28]. Thus, several authors state that triggering of apoptosis of these cells might be an important mechanism through which IFN beta reduces active lesions in the CNS of MS patients [23] [28].

Other proposed mechanisms of action of IFN beta through the TRAIL/TRAIL receptor system involve inhibiting the activation of auto-reactive T cells [29] or promoting regulatory T cells [30].

Our results show an association of the *CC* genotype in SNP rs20576 with a good response to IFN beta, reaching the Bonferroni level of significance in the joint analysis. This SNP is located in the exon 5 of *TRAILR-1*, within a conserved region of the extracellular cysteine rich domain. The *rs20576-C* allele of this variant (*683 A>C*) encodes for a *Glu228Ala* substitution close to the membrane anchoring. Interestingly, this same allele has been associated with predisposition to different cancer types [31], [32], [33], [34] as well as with an increased risk of metastasis [35], suggesting that this amino acid change would avoid the TRAIL mediated apoptosis in tumour cells. During treatment with IFN beta, the enhancement of TRAIL expression might be, on one side, inducing apoptosis in granulocytes or monocytes, preventing their demyelinating potential. On the other, it might be killing or inhibiting the activation of auto-reactive T cells, or promoting regulatory T cells. Additionally, TRAIL might be acting as a cytotoxic effector for oligodendrocytes through TRAILR-1. Supporting this, a paper by Matysiak [36] showed that TRAIL-induced apoptosis in oligodendrocytes is mediated by TRAILR-1, while apoptosis of T cells is mainly mediated by TRAILR-2 [27]. The *C* allele in rs20576 may act as a protector for oligodendrocytes in MS, avoiding the TRAIL mediated apoptosis. Our preliminary results from the expression study, with a limited sample size, do not support an effect of rs20576 on TRAILR-1 expression in PBMC subsets, as rs20576 genotypes seem to associate with the same expression levels; therefore, an effect of the *rs20576-CC* genotype in oligodendrocytes would be plausible.

In order to unravel how this amino acid substitution could modify the death signal through TRAILR-1, we performed two molecular dynamics simulations of both the “wild type” and the “mutant” receptor (Glutamic acid and Alanine at position 228 respectively), on the basis of the crystal structure of TRAILR-2. Due to its extracellular localization, our first hypothesis, supported by some other studies [35], suggested that the polymorphism could lead to a major remodelling of the key binding sites in the receptor (loops 50′s and 90′s), and therefore, to the potential loss of effectiveness in the binding between TRAIL and its receptor. Nevertheless, our results show that this amino acid substitution does not imply major structural changes in the most conserved TRAIL binding sites in TRAILR-1, so we cannot conclude that the polymorphism is actually hindering TRAIL binding. On the light of these results, we can reconsider new hypotheses by which the polymorphism may be a key intermediate in the abolition of the death signal in oligodendrocytes and other neural cells. One possibility is the alteration of the Pre-Ligand Assembly Domain (PLAD), which is an extracellular binding event, critical for TRAIL binding. TRAIL receptors are thought to be pre-assembled in the membrane by interactions through their PLAD domains, facilitating the formation of the trimer after TRAIL binding. Due to the proximity of the SNP rs20576 to the membrane, an altered signal transmission through the transmembrane region and to the cytosolic death domain could take place, avoiding the formation of the Death-Inducing Signalling Complex (DISC). In this line, a recent article has established that a Cystein residue in the domain close to the transmembrane region is essential for the pre-assembly of TRAILR-2 homodimers through interactions of the transmembrane domains [37], facilitating the death signal. Further studies are necessary to elucidate the exact mechanism.

Regarding the association of polymorphisms in *TRAIL* and *TRAIL receptors* with clinical variables in MS, only the study by Kikuchi and colleagues [38] in a Japanese population has explored this question. The authors found no association between the only SNP studied in the *TRAIL* gene, rs1131579, and the AAO. In our study, associations in the original cohort were not replicated in the validation cohort. These results are in agreement with a previous study [39], where none of the SNPs spanning the genes of *TRAIL* and its receptors emerged from the GWAS that performed AAO analyses.

In conclusion, this study provides the first evidence that a polymorphism in *TRAILR1* influences the response to IFN-beta therapy in MS. Our study discards the alteration of either the TRAIL binding site in TRAILR-1 or the expression levels of TRAILR-1 in PBMC subgroups as the underlying mechanisms. Further experiments inquiring the exact role of TRAIL and its membrane bound receptors in MS are warranted to unravel the molecular pathways of IFN beta action and to identify those patients with suboptimal response to this drug.

## Methods

### Ethics Statement

Written informed consent was obtained from patients and controls. The study was approved by the Institutional Research Ethics Committees of the respective hospitals.

(Comisión de Ética de la Investigación Málaga Nordeste from Hospital Regional Universitario Carlos Haya, Comité Ético del Hospital Universitario 12 de Octubre de Madrid, Comité Ético de Investigación Clínica del Hospital Clínico San Carlos and Comité Ético de Investigación Clínica del Instituto de Investigación Sanitaria San Carlos).

### Study Subjects

In the present study we have analysed a previously published dataset [25] with regard to the response to IFN beta treatment and clinical characteristics. In short, a total of 509 patients under treatment with IFN beta were recruited for the original cohort through the Multiple Sclerosis Unit of Hospital Regional Universitario Carlos Haya in Malaga, Spain. For the validation cohort, we selected a total of 226 patients from the Hospital 12 de Octubre (n = 51) and Hospital Clinico San Carlos (n = 175), both located in Madrid, Spain. An additional cohort of 77 MS patients treated with Glatimer Acetate was included to verify that the SNP has a true effect on response to IFN-beta treatment.

All the patients were Spanish Caucasian individuals and fulfilled the McDonald criteria for MS diagnosis [40].

The following demographic and clinical characteristics of the MS patients were assessed: sex, age, age at disease onset, disease course at present, disease duration and classification as responder or non-responder to IFN beta or Glatiramer Acetate therapy regarding relapses and disability progression during the two-year follow-up period after treatment onset. These demographic and clinical characteristics of the MS patients are summarised in Table 3.

**Table 3:** Demographic and clinical characteristics of the MS patients.

Characteristics	Original cohort	Validation cohort	Glatiramer Acetate treated patients
Gender (%):			
Female	352(69.2%)	149(65.9%)	53(68.8%)
Male	157(30.8%)	77(34.1%)	24(31.2%)
Age (years)	43.75±11.11 (15–76)	41.27±9.21 (20–72)	42.29±12.34 (19–73)
Mean age at onset (years)	29.79±9.71 (7–61)	28.79±7.74 (8–53)	30.15±10.56 (4–68)
Time of disease	14.05±8.22 (1–40)	13.11±7.71 (1–49)	12.56±8.46 (2–41)
Disease Course:			
Relapsing-Remiting	383(75.2%)	209(92.5%)	64(83.1%)
Secondary Progressive	126(24.8%)	17(7.5%)	13(16.9%)
Non Responders to therapy (%)	296 (58.2%)	122 (54.0%)	48 (62.3%)

Quantitative data are presented as mean ± standard deviation (minimum–maximum).

### Definition of Response to Interferon Beta Therapy

Clinical criteria during the first two years of therapy were assessed to classify patients as responders and nonresponders. We based our response criteria in the “G” criteria from an article by Rio and colleagues [5]. Briefly, patients were classified as responders if they had no relapses and no progression of the disease during the follow-up period and as nonresponders or suboptimal responders if they had one or more relapses or any progression of the disease during the follow-up period (an increase of at least 1.5 points in the EDSS for patients with a baseline EDSS score lower than 1, and 1 point increase in the EDSS for patients with a baseline EDSS score equal or higher than 1).

### Detection of Neutralizing Antibodies

Patients were tested for Neutralizing Antibodies (NABs) against IFN beta, which could be a confounding factor for the response to treatment. The presence of NABs in serum samples was determined after 12 and 24 months of treatment by the interferon-induced inhibition of virus cytopathic effect on human cells in culture (murine encephalomyocarditis virus and A-549 cells), following the World Health Organization (WHO) recommendations, as previously described [41], [42]. The neutralization titre of a serum sample was calculated according to Kawade et al. [43] and expressed in 10-fold reduction units per millilitre (TRU/mL). Titres ≥20 TRU/mL were considered as positive.

### SNP Identification and Selection

Tag-SNPs spanning the genes encoding *TRAIL* (mapped at chromosome 3q26) and its four receptors (*TRAILR-1, TRAILR-2, TRAILR-3* and *TRAILR-4,* mapped at chromosome 8p21–22) were selected using the web tool “SYSNPs” (www.sysnps.org). Flanking regions of 2000 bp upstream and 500 bp downstream were included. A minor allele frequency of at least 0.1 and a minimum r^2^ coefficient of 0.8 were used to select 54 Tag-SNPs. In addition, we also selected five exonic SNPs in the *TRAIL* gene, corresponding to rs6763816 and rs11545817 in exon 1, rs16845759 in exon 2, rs4491934 in exon 3 and rs1131579 in the 3′ UTR of exon 5.

### Genotyping

Genomic DNA was extracted from peripheral blood nucleated cells using the Genomic DNA Purification Kit® (Gentra Systems Inc, Minneapolis, MN, USA). All polymorphisms were genotyped using TaqMan assays (AppliedBiosystems, Inc., Foster City, CA, USA) and the OpenArray Platform (BioTrove, Woburn, MA, USA) following the protocols recommended by manufacturers. In short, reactions were performed in 3072 through-hole arrays under the following conditions: 93°C for 10 minutes, followed by 50 cycles of 95°C for 45 seconds, 94°C for 13 seconds and 53°C for 134 seconds. The DNA concentration used was 50 ng/µL.

### Separation of Monocytes and CD4+ and CD8+ T Lymphocytes

Peripheral blood mononuclear cells (PBMC) were isolated from whole blood by density gradient centrifugation. Monocytes (CD14+), CD8+ T cells and CD4+ T cells were isolated by positive selection with immunomagnetic sorting (Miltenyi Biotec GmbH, Bergisch Gladbach, Germany), following the manufacturer protocols. Purity of each cellular subset was assessed by flow cytometry in a FACS CANTO II cytometer.

After separation, cell subsets were cultured in 96 well plates with RPMI medium supplemented with 10% FBS, 1.3% Hepes, 50 µg/mL Gentamicin and 2 mM L-Glutamine for one day. Medium was changed the following day for the same medium lacking FBS, and after 1 hour, cells were stimulated with 20 UI/mL of IFN beta (Avonex®, Biogen Idec). Cells were collected 24 hours after the stimulation.

### RNA Isolation, Primers Design and RT-qPCR

Total RNA was isolated using the Phenol-Chloroform method [44] Yield of the isolation protocol and quality of product was assessed by measuring with Nanodrop 2000 Spectrophotometer (Thermo Fisher Scientific Inc).

For cDNA synthesis, we performed a two-step RT-PCR. For each sample, two reactions were performed. A mix of 1 µg of RNA and 1 µL of random Primer p(dN)6 (Roche Diagnostic GmbH) in a total volume of 10 µL was incubated for 10 minutes at 70°C. After incubation, 1 µL dNTP, 0.5 µL Protector RNase Inhibitor (Roche Diagnostic GmbH), 1 µL M-MLV Reverse Transcriptase (Sigma-Aldrich), 1 µL 10× buffer and 6.5 µL DEPC-treated water were added (Sigma-Aldrich). The cDNA was stored at −80°C until use.

Primers for TRAILR-1 were design using Primer3 software [45]. To ensure that primer pairs amplified the specific products, and to determine the optimal annealing temperature, conventional PCR using temperatures from 57°C to 63°C were performed, and products were visualised in an agarose gel. Molecular Weight Marker IX (Roche Diagnostic GmbH) was included in the electrophoresis in order to determine the sizes of different amplified products. This way, we determined that annealing at 57°C was optimal for TRAILR1, and at 60°C to amplify the reference gene GAPDH.

qPCR was performed in duplicate in a Rotor Gene® Q thermocycler (Qiagen). A total of 20 µL reaction mix was prepared: 6.4 µL DEPC treated water, 1 µL 20 mM primers (forward and reverse), 10.6 µL Quantitec® SYBR® Green PCR Master Mix (Qiagen) and 2 µL cDNA. The PCR program consisted in a step of 15 minutes at 95°C, followed by 40 cycles of 95°C for 30 seconds, 57 or 60°C for 30 seconds and 72°C for 30 seconds. A melting step was run, from 65 to 95°C, increasing 0.5°C every 5 seconds.

### Statistical Analysis

Statistical power was calculated using QUANTO 1.2.4 (http://hydra.usc.edu/gxe) with the following configuration: Population risk = 0.5; Inheritance model = additive; Minor allele frequency = 0.1.

Statistical analysis was performed using the SPSS software (version 11.5.1) and the SNPassoc R package (R software version 2.10.0) [46].

Deviations from Hardy-Weinberg Equilibrium were tested using an exact test as described by Wigginton and colleagues [47] and multiple testing corrections were carried out.

Even after logarithmic transformation of the AAO was used, we were not able to meet the normality assumption of the regression model, so non parametric tests were used to find associations between individual SNPs and AAO. To test if any individual SNP was associated with the response to IFN beta, genotype frequencies were compared using a likelihood ratio test under four different genetic models (Codominant, Dominant, Recessive and Additive). P values lower than 0.05 were considered to be statistically significant. Logistic regression models were used to estimate crude odds ratios (ORs) as well as 95% confidence intervals (95% CI). To avoid false-positive results due to multiple testing, we applied the Bonferroni correction that is robust against positive dependence.

To compare relative expression of TRAIL-R1 in cell subsets in patients stratified by the rs20576 genotype (A/− vs CC) we performed a Mann Whitney test.

### Molecular Dynamics Simulations

An initial alignment of the amino acid sequence of TRAILR-1 and TRAILR-2 was performed using T-coffee [48] and the mutation of the selected residues was done with PyMOL (www.pymol.org). Our two systems have been built on the basis of the crystal structure of the death receptor 5 (DR5) bound to TRAIL (PDB 1D0G) and they are comprised by B and D chains of the ligand and the T chain of the receptor. This composition covers the surface of interaction between one monomer of TRAILR-2 and its ligand. The high conservation in the amino acid sequence and secondary structure of both receptors has allowed us to use a slightly modified structure of TRAILR-2. The wild type structure was only modified in the position G168K (see Results) whereas the structure carrying the polymorphism was modified in the positions G168K and E177A.

In order to study the dynamic behaviour of the systems, we carried out two all-atom molecular dynamics (MD) simulations. Coordinates and topology files were generated using the program LEaP of the AMBER package. In this process, the cysteine rich domains were specified in order to maintain the integrity of the disulfide bonds during the simulation. The complexes were solvated with a 12 Å box of TIP3P water molecules [49] and neutralized with counterions. The Particle Mesh Ewald method [50] was used to treat long-range electrostatic interactions. The systems were minimized using both steepest descent and conjugate gradient methods. Afterwards, the systems were heated to 300 K and equilibrated for 100 ps with Cα restraints. These restraints began with an initial value of 20 kcal·mol^−1^·Å^−2^ and decreased by 5 kcal·mol^−1^·Å^−2^ in five 20 ps steps to the unrestrained state. The MD simulations were run for 10 ns at 300 K using the sander program of the Amber package and the Amber ff03 force field [51]. MD trajectories were processed with ptraj module of AMBER9.

## Supporting Information

Table S1 Genotype frequencies obtained from Original and Validation cohorts.
(DOC)

Table S2 Genotype frequencies obtained from the joint analysis, stratified by response to IFN beta.
(DOC)

Table S3 Genotype frequencies for rs20576 according to response to IFN beta treatment and disease course.
(DOC)

Table S4 Genotype frequencies for rs20576 according to response to Glatiramer Acetate.
(DOC)
