# Supplementary material for: Candidate Gene Study of TRAIL and TRAIL Receptors: Association with Response to Interferon Beta Therapy in Multiple Sclerosis Patients
Source: PLoS One. 2013 Apr 29;8(4):e62540. doi: 10.1371/journal.pone.0062540 (PMC3639207; doi:10.1371/journal.pone.0062540)
Supplement: Table S1 — Genotype frequencies obtained from Original and Validation cohorts. (DOC) [file pone.0062540.s001.doc]

**Table S1. Genotype frequencies obtained from Original and Validation cohorts.**

| SNP ID | Gene | Chr. | Location | Allele  1 > 2 | Original Cohort (Málaga) | | | Validation Cohort (Madrid) | | |
| --- | --- | --- | --- | --- | --- | --- | --- | --- | --- | --- |
|  |  |  |  |  | MS patients (n = 628) (%) | | | MS patients (n = 295) (%) | | |
|  |  |  |  |  | 11 | 12 | 22 | 11 | 12 | 22 |
| rs3136594 | TRAIL | 3 | Intronic | C>T | 260 (42.2%) | 297 (48.2%) | 59 (9.6%) | 120 (42.3%) | 133 (46.8%) | 31 (10.9%) |
| rs4894559 | TRAIL | 3 | Intronic | G>A | 400 (66.3%) | 173 (28.7%) | 30 (3.5%) | 167 (59.2%) | 108 (38.3%) | 7 (2.5%) |
| rs231983 | TRAIL | 3 | Intronic | A>C | 305 (48.6%) | 269 (42.9%) | 53 (8.5%) | 132 (45.2%) | 130 (44.5%) | 30 (10.3%) |
| rs179777 | TRAIL | 3 | Intronic | A>G | 446 (71.9%) | 167 (26.9%) | 7 (1.1%) | 205 (72.2%) | 70 (24.6%) | 9 (3.2%) |
| rs3136581 | TRAIL | 3 | 5' Upstream | C>T | 391 (62.7%) | 205 (32.9%) | 28 (4.5%) | 197 (67.7%) | 80 (27.5%) | 14 (4.8%) |
| rs6763816 | TRAIL | 3 | Exon 1, NSC | C>T | 616 (99.5%) | 3 (0.5%) | 0 (0.0%) | 287 (100%) | 0 (0.0%) | 0 (0.0%) |
| rs16845759 | TRAIL | 3 | Exon 2, NSC | G>T | 611 (97.9%) | 13 (2.1%) | 0 (0.0%) | 277 (97.5%) | 7 (2.5%) | 0 (0.0%) |
| rs4491934 | TRAIL | 3 | Exon 3, NSC | G>A | 604 (99.7%) | 2 (0.3%) | 0 (0.0%) | 277 (98.9%) | 3 (1.1%) | 0 (0.0%) |
| rs1823227 | TRAIL | 3 | Intronic | T>G | 272 (43.9%) | 281 (45.3%) | 67 (10.8%) | 131 (45.0%) | 132 (45.4%) | 28 (9.6%) |
| rs3136587 | TRAIL | 3 | Intronic | T>C | 471 (75.2%) | 140 (22.4%) | 15 (2.4%) | 215 (73.6%) | 70 (24.0%) | 7 (2.4%) |
| rs1131579 | TRAIL | 3 | Exon 5, 3´UTR | G>A | 586 (99.8%) | 1 (0.2%) | 0 (0.0%) | 274 (100%) | 0 (0.0%) | 0 (0.0%) |
| rs11545817 | TRAIL | 3 | Exon 1, NSC | G>A | 626 (100%) | 0 (0.0%) | 0 (0.0%) | 274 (100%) | 0 (0.0%) | 0 (0.0%) |
| rs2230229 | TRAILR-1 | 8 | Exon 10, NSC | A>G | 441 (71.9%) | 164 (26.8%) | 8 (1.3%) | 213 (72.9%) | 73 (25.0%) | 6 (2.1%) |
| rs11775256 | TRAILR-1 | 8 | Intronic | C>T | 365 (59.9%) | 212 (34.8%) | 32 (5.3%) | 175 (61.0%) | 94 (32.8%) | 18 (6.3%) |
| rs11780345 | TRAILR-1 | 8 | Intronic | T>C | 280 (45.4%) | 268 (43.4%) | 69 (11.2%) | 140 (48.1%) | 121 (41.6%) | 30 (10.3%) |
| rs6557627 | TRAILR-1 | 8 | Intronic | C>G | 418 (67.3%) | 181 (29.1%) | 22 (3.5%) | 190 (66.4%) | 83 (29.0%) | 13 (4.5%) |
| rs2235126 | TRAILR-1 | 8 | Intronic | C>T | 297 (48.0%) | 275 (44.4%) | 47 (7.6%) | 146 (50.3%) | 120 (41.4%) | 24 (8.3%) |
| rs10097540 | TRAILR-1 | 8 | Intronic | C>A | 370 (61.8%) | 203 (33.9%) | 26 (4.3%) | 180 (64.5%) | 86 (30.8%) | 13 (4.7%) |
| rs4872077 | TRAILR-1 | 8 | Intronic | T>C | 342 (55.4%) | 231 (37.1%) | 44 (7.1%) | 145 (50.9%) | 111 (38.9%) | 29 (10.2%) |
| rs20576 | TRAILR-1 | 8 | Exon 5, NSC | A>C | 371 (59.4%) | 221 (35.4%) | 33 (5.3%) | 166 (57.8%) | 105 (36.6%) | 16 (5.6%) |
| rs4242392 | TRAILR-1 | 8 | Intronic | T>C | 372 (59.7%) | 215 (34.5%) | 36 (5.8%) | 179 (61.3%) | 99 (33.9%) | 14 (4.8%) |
| rs6995408 | TRAILR-1 | 8 | Intronic | G>A | 142 (23.7%) | 310 (51.8%) | 147 (24.5%) | 70 (24.9%) | 153 (54.4%) | 58 (20.6%) |
| rs4526369 | TRAILR-1 | 8 | Intronic | A>G | 336 (54.3%) | 248 (40.1%) | 35 (5.7%) | 170 (58.2%) | 104 (35.6%) | 18 (6.2%) |
| rs11785328 | TRAILR-1 | 8 | Intronic | C>T | 330 (53.0%) | 250 (40.1%) | 43 (6.9%) | 151 (53.2%) | 113 (39.8%) | 20 (7.0%) |
| rs13255394 | TRAILR-1 | 8 | 5' Upstream | T>C | 163 (30.6%) | 231 (43.3%) | 139 (26.1%) | 54 (19.1%) | 160 (56.5%) | 69 (24.4%) |
| rs11779484 | TRAILR-1 | 8 | Intronic | T>C | 551 (88.7%) | 67 (10.8%) | 3 (0.5%) | 255 (87.3%) | 34 (11.6%) | 3 (1.0%) |
| rs6557628 | TRAILR-1 | 8 | Intronic | T>G | 405 (65.7%) | 183 (29.7%) | 28 (4.5%) | 198 (70.5%) | 76 (27.0%) | 7 (2.5%) |
| rs1047275 | TRAILR-2 | 8 | 3' UTR | C>G | 170 (27.3%) | 327 (52.5%) | 126 (20.2%) | 79 (27.1%) | 141 (48.5%) | 71 (24.4%) |
| rs6557609 | TRAILR-2 | 8 | Intronic | C>T | 478 (76.6%) | 134 (21.5%) | 12 (1.9%) | 216 (75.3%) | 66 (23.0%) | 5 (1.7%) |
| rs7834266 | TRAILR-2 | 8 | Intronic | C>T | 250 (40.1%) | 284 (45.5%) | 90 (14.4%) | 115 (39.5%) | 137 (47.1%) | 39 (13.4%) |
| rs1001793 | TRAILR-2 | 8 | Intronic | G>A | 290 (46.8%) | 272 (43.9%) | 57 (9.2%) | 138 (48.6%) | 113 (39.8%) | 33 (11.6%) |
| rs13270480 | TRAILR-2 | 8 | Intronic | T>A | 313 (51.7%) | 248 (40.9%) | 45 (7.4%) | 155 (56.2%) | 105 (38.0%) | 16 (5.8%) |
| rs7843721 | TRAILR-2 | 8 | Intronic | G>T | 457 (73.9%) | 143 (23.1%) | 18 (2.9%) | 203 (73.0%) | 63 (22.7%) | 12 (4.3%) |
| rs4424253 | TRAILR-2 | 8 | Intronic | C>T | 447 (72.7%) | 150 (24.4%) | 18 (2.9%) | 197 (68.4%) | 79 (27.4%) | 12 (4.2%) |
| rs11135693 | TRAILR-2 | 8 | Intronic | C>A | 245 (39.5%) | 290 (46.7%) | 86 (13.8%) | 118 (40.5%) | 150 (51.5%) | 23 (7.9%) |
| rs4460370 | TRAILR-2 | 8 | Intronic | C>T | 269 (45.1%) | 256 (43.0%) | 71 (11.9%) | 130 (46.4%) | 117 (41.8%) | 33 (11.8%) |
| rs11135696 | TRAILR-3 | 8 | 5' UTR | G>A | 394 (63.7%) | 207 (33.4%) | 18 (2.9%) | 189 (65.4%) | 94 (32.5%) | 6 (2.1%) |
| rs4518666 | TRAILR-3 | 8 | Intronic | T>C | 291 (46.6%) | 272 (43.6%) | 61 (9.8%) | 124 (43.2%) | 123 (42.9%) | 40 (13.9%) |
| rs4872052 | TRAILR-3 | 8 | Intronic | T>C | 506 (81.2%) | 109 (17.5%) | 8 (1.3%) | 216 (74.0%) | 63 (21.6%) | 13 (4.5%) |
| rs4871846 | TRAILR-3 | 8 | Intronic | C>G | 267 (42.9%) | 276 (44.3%) | 80 (12.8%) | 105 (36.2%) | 131 (45.2%) | 54 (18.6%) |
| rs7008760 | TRAILR-3 | 8 | Intronic | C>G | 171 (28.5%) | 279 (46.4%) | 151 (25.1%) | 64 (23.4%) | 130 (47.6%) | 79 (28.9%) |
| rs12681513 | TRAILR-3 | 8 | Intronic | G>A | 434 (71.5%) | 167 (27.5%) | 6 (1.0%) | 204 (72.1%) | 69 (24.4%) | 10 (3.5%) |
| rs4077341 | TRAILR-3 | 8 | Intronic | T>G | 272 (43.5%) | 270 (43.1%) | 84 (13.4%) | 117 (40.2%) | 128 (44.0%) | 46 (15.8%) |
| rs12546238 | TRAILR-3 | 8 | Intronic | C>T | 506 (81.1%) | 111 (17.8%) | 7 (1.1%) | 228 (79.4%) | 56 (19.5%) | 3 (1.0%) |
| rs6557616 | TRAILR-3 | 8 | Exon 1, NSC | C>G | 377 (60.7%) | 223 (35.9%) | 21 (3.4%) | 174 (59.6%) | 111 (38.0%) | 7 (2.4%) |
| rs9314261 | TRAILR-3 | 8 | Intronic | G>A | 388 (73.2%) | 126 (23.8%) | 16 (3.0%) | 191 (67.5%) | 84 (29.7%) | 8 (2.8%) |
| rs7957 | TRAILR-4 | 8 | 3' UTR | T>C | 427 (68.4%) | 177 (28.4%) | 20 (3.2%) | 207 (71.1%) | 69 (23.7%) | 15 (5.2%) |
| rs7011559 | TRAILR-4 | 8 | Intronic | A>G | 428 (68.9%) | 174 (28.0%) | 19 (3.1%) | 192 (66.4%) | 86 (29.8%) | 11 (3.8%) |
| rs6557618 | TRAILR-4 | 8 | Intronic | T>A | 305 (49.4%) | 267 (43.2%) | 46 (7.4%) | 147 (51.8%) | 106 (37.3%) | 31 (10.9%) |
| rs1133782 | TRAILR-4 | 8 | Exon 7, NSC | C>T | 240 (38.8%) | 296 (47.8%) | 83 (13.4%) | 126 (43.0%) | 122 (41.6%) | 45 (15.4%) |
| rs3924519 | TRAILR-4 | 8 | Intronic | T>C | 305 (50.2%) | 256 (42.2%) | 46 (7.6%) | 133 (47.2%) | 118 (41.8%) | 31 (11.0%) |
| rs4871850 | TRAILR-4 | 8 | Intronic | A>G | 313 (50.2%) | 249 (40.0%) | 61 (9.8%) | 154 (53.7%) | 110 (38.3%) | 23 (8.0%) |
| rs7014131 | TRAILR-4 | 8 | Intronic | T>A | 384 (61.7%) | 204 (32.8%) | 34 (5.5%) | 189 (65.2%) | 87 (30.0%) | 14 (4.8%) |
| rs7462795 | TRAILR-4 | 8 | Intronic | C>T | 479 (77.3%) | 133 (21.5%) | 8 (1.3%) | 225 (79.8%) | 52 (18.4%) | 5 (1.8%) |

Abbreviations: SNP ID, SNP identification; Chr, chromosome; 1>2, major>minor allele; NSC: Non Synonymous Coding.
