# Supplementary material for: Candidate Gene Study of TRAIL and TRAIL Receptors: Association with Response to Interferon Beta Therapy in Multiple Sclerosis Patients
Source: PLoS One. 2013 Apr 29;8(4):e62540. doi: 10.1371/journal.pone.0062540 (PMC3639207; doi:10.1371/journal.pone.0062540)
Supplement: Table S2 — Genotype frequencies obtained from the joint analysis, stratified by response to IFN beta. (DOC) [file pone.0062540.s002.doc]

**Table S2. Genotype frequencies obtained from the joint analysis, stratified by response to IFN beta.**

| SNP ID | Gene | Chr. | Location | Allele  1 > 2 | Responders (n = 317) (%) | | | Non Responders (n = 418) (%) | | |
| --- | --- | --- | --- | --- | --- | --- | --- | --- | --- | --- |
|  |  |  |  |  | 11 | 12 | 22 | 11 | 12 | 22 |
| rs3136594 | TRAIL | 3 | Intronic | C>T | 121 (39.2) | 163 (52.8) | 25 (8.1) | 171 (42.3) | 191 (47.3) | 42 (10.4) |
| rs4894559 | TRAIL | 3 | Intronic | G>A | 196 (64.7) | 93 (30.7) | 14 (4.6) | 254 (63.7) | 126 (31.6) | 19 (4.8) |
| rs231983 | TRAIL | 3 | Intronic | A>C | 156 (49.4) | 134 (42.4) | 26 (8.2) | 205 (49.2) | 173 (41.5) | 39 (9.4) |
| rs179777 | TRAIL | 3 | Intronic | A>G | 215 (69.6) | 87 (28.2) | 7 (2.3) | 302 (74.0) | 98 (24.0) | 8 (2.0) |
| rs3136581 | TRAIL | 3 | 5' Upstream | C>T | 207 (65.9) | 95 (30.3) | 12 (3.8) | 259 (62.6) | 136 (32.9) | 19 (4.6) |
| rs6763816 | TRAIL | 3 | Exon 1, NSC | C>T | 309 (99.4) | 2 (0.6) | 0 (0.0) | 410 (99.8) | 1 (0.2) | 0 (0.0) |
| rs16845759 | TRAIL | 3 | Exon 2, NSC | G>T | 299 (96.1) | 12 (3.9) | 0 (0.0) | 405 (98.3) | 7 (1.7) | 0 (0.0) |
| rs4491934 | TRAIL | 3 | Exon 3, NSC | G>A | 300 (98.7) | 4 (1.3) | 0 (0.0) | 395 (99.7) | 1 (0.3) | 0 (0.0) |
| rs1823227 | TRAIL | 3 | Intronic | T>G | 129 (41.5) | 153 (49.2) | 29 (9.3) | 183 (44.3) | 182 (44.1) | 48 (11.6) |
| rs3136587 | TRAIL | 3 | Intronic | T>C | 232 (73.7) | 78 (24.8) | 5 (1.6) | 316 (75.8) | 88 (21.1) | 13 (3.1) |
| rs1131579 | TRAIL | 3 | Exon 5, 3´UTR | G>A | 299 (100) | 0 (0.0) | 0 (0.0) | 387 (99.7) | 1 (0.3) | 0 (0.0) |
| rs11545817 | TRAIL | 3 | Exon 1, NSC | G>A | 315 (100) | 0 (0.0) | 0 (0.0) | 415 (100) | 0 (0.0) | 0 (0.0) |
| rs2230229 | TRAILR-1 | 8 | Exon 10, NSC | A>G | 220 (71.0) | 85 (27.4) | 5 (1.6) | 305 (74.6) | 100 (24.4) | 4 (1.0) |
| rs11775256 | TRAILR-1 | 8 | Intronic | C>T | 183 (60.0) | 104 (34.1) | 18 (5.9) | 245 (60.5) | 143 (35.3) | 17 (4.2) |
| rs11780345 | TRAILR-1 | 8 | Intronic | T>C | 145 (46.2) | 135 (43.0) | 34 (10.8) | 185 (45.1) | 181 (44.1) | 44 (10.7) |
| rs6557627 | TRAILR-1 | 8 | Intronic | C>G | 198 (63.5) | 101 (32.4) | 13 (4.2) | 282 (69.3) | 112 (27.5) | 13 (3.2) |
| rs2235126 | TRAILR-1 | 8 | Intronic | C>T | 160 (51.3) | 131 (42.0) | 21 (6.7) | 194 (47.1) | 181 (43.9) | 37 (9.0) |
| rs10097540 | TRAILR-1 | 8 | Intronic | C>A | 179 (59.3) | 104 (34.4) | 19 (6.3) | 236 (60.2) | 143 (36.5) | 13 (3.3) |
| rs4872077 | TRAILR-1 | 8 | Intronic | T>C | 170 (54.3) | 122 (39.0) | 21 (6.7) | 212 (52.5) | 156 (38.6) | 36 (8.9) |
| rs20576 | TRAILR-1 | 8 | Exon 5, NSC | A>C | 182 (57.8) | 109 (34.6) | 24 (7.6) | 252 (60.3) | 155 (37.2) | 10 (2.4) |
| rs4242392 | TRAILR-1 | 8 | Intronic | T>C | 180 (57.3) | 116 (36.9) | 18 (5.7) | 253 (61.1) | 140 (33.8) | 21 (5.1) |
| rs6995408 | TRAILR-1 | 8 | Intronic | G>A | 74 (24.6) | 156 (51.8) | 71 (23.6) | 91 (22.9) | 206 (51.9) | 100 (25.2) |
| rs4526369 | TRAILR-1 | 8 | Intronic | A>G | 185 (58.7) | 114 (36.2) | 16 (5.1) | 221 (53.8) | 159 (38.7) | 31 (7.5) |
| rs11785328 | TRAILR-1 | 8 | Intronic | C>T | 175 (56.1) | 122 (39.1) | 15 (4.8) | 209 (51.2) | 165 (40.4) | 34 (8.3) |
| rs13255394 | TRAILR-1 | 8 | 5' Upstream | T>C | 82 (30.0) | 132 (48.4) | 59 (21.6) | 91 (24.9) | 177 (48.4) | 98 (26.8) |
| rs11779484 | TRAILR-1 | 8 | Intronic | T>C | 277 (88.2) | 33 (10.5) | 4 (1.3) | 363 (87.3) | 51 (12.3) | 2 (0.5) |
| rs6557628 | TRAILR-1 | 8 | Intronic | T>G | 201 (65.3) | 94 (30.5) | 13 (4.2) | 277 (68.4) | 114 (28.1) | 14 (3.5) |
| rs1047275 | TRAILR-2 | 8 | 3' UTR | C>G | 78 (24.9) | 166 (53.0) | 69 (22.0) | 118 (28.5) | 202 (48.8) | 94 (22.7) |
| rs6557609 | TRAILR-2 | 8 | Intronic | C>T | 231 (73.8) | 73 (23.3) | 9 (2.9) | 323 (78.4) | 82 (19.9) | 7 (1.7) |
| rs7834266 | TRAILR-2 | 8 | Intronic | C>T | 115 (36.5) | 156 (49.5) | 44 (14.0) | 177 (42.9) | 181 (43.8) | 55 (13.3) |
| rs1001793 | TRAILR-2 | 8 | Intronic | G>A | 141 (45.6) | 141 (45.6) | 27 (8.7) | 193 (47.4) | 163 (40.0) | 51 (12.5) |
| rs13270480 | TRAILR-2 | 8 | Intronic | T>A | 155 (51.3) | 130 (43.0) | 17 (5.6) | 215 (54.0) | 148 (37.2) | 35 (8.8) |
| rs7843721 | TRAILR-2 | 8 | Intronic | G>T | 222 (72.1) | 75 (24.4) | 11 (3.6) | 302 (74.9) | 93 (23.1) | 8 (2.0) |
| rs4424253 | TRAILR-2 | 8 | Intronic | C>T | 227 (73.2) | 74 (23.9) | 9 (2.9) | 289 (70.8) | 103 (25.2) | 16 (3.9) |
| rs11135693 | TRAILR-2 | 8 | Intronic | C>A | 118 (37.8) | 161 (51.6) | 33 (10.6) | 178 (43.0) | 184 (44.4) | 52 (12.6) |
| rs4460370 | TRAILR-2 | 8 | Intronic | C>T | 137 (45.4) | 133 (44.0) | 32 (10.6) | 177 (44.4) | 168 (42.1) | 54 (13.5) |
| rs11135696 | TRAILR-3 | 8 | 5' UTR | G>A | 198 (63.5) | 108 (34.6) | 6 (1.9) | 257 (62.7) | 141 (34.4) | 12 (2.9) |
| rs4518666 | TRAILR-3 | 8 | Intronic | T>C | 149 (47.8) | 128 (41.0) | 35 (11.2) | 187 (45.4) | 177 (43.0) | 48 (11.7) |
| rs4872052 | TRAILR-3 | 8 | Intronic | T>C | 253 (80.3) | 52 (16.5) | 10 (3.2) | 315 (76.3) | 87 (21.1) | 11 (2.7) |
| rs4871846 | TRAILR-3 | 8 | Intronic | C>G | 132 (42.0) | 141 (44.9) | 41 (13.1) | 171 (41.4) | 171 (41.4) | 71 (17.2) |
| rs7008760 | TRAILR-3 | 8 | Intronic | C>G | 86 (28.5) | 135 (44.7) | 81 (26.8) | 105 (26.9) | 183 (46.9) | 102 (26.2) |
| rs12681513 | TRAILR-3 | 8 | Intronic | G>A | 210 (68.9) | 90 (29.5) | 5 (1.6) | 298 (74.1) | 98 (24.4) | 6 (1.5) |
| rs4077341 | TRAILR-3 | 8 | Intronic | T>G | 134 (42.7) | 136 (43.3) | 44 (14.0) | 177 (42.4) | 180 (43.2) | 60 (14.4) |
| rs12546238 | TRAILR-3 | 8 | Intronic | C>T | 251 (80.2) | 59 (18.8) | 3 (1.0) | 330 (80.1) | 78 (18.9) | 4 (1.0) |
| rs6557616 | TRAILR-3 | 8 | Exon 1, NSC | C>G | 186 (59.2) | 117 (37.3) | 11 (3.5) | 246 (59.7) | 155 (37.6) | 11 (2.7) |
| rs9314261 | TRAILR-3 | 8 | Intronic | G>A | 199 (70.8) | 76 (27.0) | 6 (2.1) | 240 (67.2) | 105 (29.4) | 12 (3.4) |
| rs7957 | TRAILR-4 | 8 | 3' UTR | T>C | 211 (67.4) | 92 (29.4) | 10 (3.2) | 297 (71.4) | 105 (25.2) | 14 (3.4) |
| rs7011559 | TRAILR-4 | 8 | Intronic | A>G | 212 (68.4) | 87 (28.1) | 11 (3.5) | 276 (66.8) | 124 (30.0) | 13 (3.1) |
| rs6557618 | TRAILR-4 | 8 | Intronic | T>A | 150 (48.5) | 134 (43.4) | 25 (8.1) | 205 (50.4) | 168 (41.3) | 34 (8.4) |
| rs1133782 | TRAILR-4 | 8 | Exon 7, NSC | C>T | 119 (37.9) | 149 (47.5) | 46 (14.6) | 169 (41.1) | 190 (46.2) | 52 (12.7) |
| rs3924519 | TRAILR-4 | 8 | Intronic | T>C | 155 (50.7) | 120 (39.2) | 31 (10.1) | 185 (46.3) | 176 (44.0) | 39 (9.8) |
| rs4871850 | TRAILR-4 | 8 | Intronic | A>G | 159 (51.1) | 123 (39.5) | 29 (9.3) | 210 (51.0) | 166 (40.3) | 36 (8.7) |
| rs7014131 | TRAILR-4 | 8 | Intronic | T>A | 201 (64.4) | 95 (30.4) | 16 (5.1) | 253 (61.3) | 138 (33.4) | 22 (5.3) |
| rs7462795 | TRAILR-4 | 8 | Intronic | C>T | 241 (77.5) | 65 (20.9) | 5 (1.6) | 312 (76.8) | 88 (21.7) | 6 (1.5) |

Abbreviations: SNP ID, SNP identification; Chr, chromosome; 1>2, major>minor allele; NSC: Non Synonymous Coding.
