# Supplementary material for: Candidate Gene Study of TRAIL and TRAIL Receptors: Association with Response to Interferon Beta Therapy in Multiple Sclerosis Patients
Source: PLoS One. 2013 Apr 29;8(4):e62540. doi: 10.1371/journal.pone.0062540 (PMC3639207; doi:10.1371/journal.pone.0062540)
Supplement: Table S3 — Genotype frequencies for rs20576 according to response to IFN beta treatment and disease course. (DOC) [file pone.0062540.s003.doc]

**Table S3. Genotype frequencies for rs20576 according to response to IFN beta treatment and disease course.**

| Disease course | Response to IFN beta | rs20576 A/- (%) | rs20576 CC (%) | p value | OR (95% CI) |
| --- | --- | --- | --- | --- | --- |
| RRMS | Responders | 246 (92.8) | 19 (7.2) | 3.15x10^-3^ | 0.29 (0.12-0.70) |
|  | Non Responders | 315 (97.8) | 7 (2.2) |  |  |
| SPMS | Responders | 45 (90.0) | 5 (10.0) | 0.0965 | 0.29 (0.07-1.28) |
|  | Non Responders | 92 (96.8) | 3 (3.2) |  |  |

Abbreviations: RRMS, Relapsing-Remitting patients; SPMS, Secondary Progressive patients; OR, Odds ratio; CI, Confidence Interval.
