# Supplementary material for: Candidate Gene Study of TRAIL and TRAIL Receptors: Association with Response to Interferon Beta Therapy in Multiple Sclerosis Patients
Source: PLoS One. 2013 Apr 29;8(4):e62540. doi: 10.1371/journal.pone.0062540 (PMC3639207; doi:10.1371/journal.pone.0062540)
Supplement: Table S4 — Genotype frequencies for rs20576 according to response to Glatiramer Acetate. (DOC) [file pone.0062540.s004.doc]

**Table S4. Genotype frequencies for rs20576 according to response to Glatiramer Acetate.**

| rs20576 genotype | Responders (%) | Non responders (%) | Inheritance Model | p value | OR (95% C.I.) |
| --- | --- | --- | --- | --- | --- |
| AA | 13 (44.8) | 27 (56.3) | Codominant [AC vs AA] Codominant [CC vs AA] Dominant [AA vs (AC + CC)] Recessive [CC vs (AA+AC)] Log-Additive [AA=0; AC=1; CC=2] | 0.6050 0.6050 0.3308 0.8705 0.4451 | 0.60 (0.22-1.65) 0.72 (0.17-3.01) 0.63 (0.25-1.60) 0.89 (0.23-3.47) 0.78 (0.41-1.49) |
| AC | 12 (41.4) | 15 (31.3) |
| CC | 4 (13.8) | 6 (12.5) |

Abbreviations: OR, Odds ratio; CI, Confidence Interval.
